# Diagnostic criteria and outcome measures in randomized clinical trials on carpal tunnel syndrome: a systematic review

**DOI:** 10.1590/1516-3180.2022.0086.07022023

**Published:** 2023-04-17

**Authors:** Rafael Luz Sousa, Vinicius Ynoe de Moraes, Alexandre Figueiredo Zobiole, Luis Renato Nakachima, João Carlos Belloti

**Affiliations:** I MD. Hand Surgeon and Master's Student, Discipline of Hand and Upper Limb Surgery, Universidade Federal de São Paulo (UNIFESP), São Paulo (SP), Brazil.; II MD, PhD. Hand Surgeon, Discipline of Hand and Upper Limb Surgery, Universidade Federal de São Paulo (UNIFESP), São Paulo (SP), Brazil; and Hand Surgeon, Hand Surgery Service, Hospital Alvorada Moema, United Health, São Paulo (SP), Brazil.; United Health, Hospital Alvorada Moema, São Paulo, SP, Brazil; III MD. Orthopedist and Fellow of shoulder and elbow at the Sports Traumatology Center, Universidade Federal de São Paulo (UNIFESP), São Paulo (SP), Brazil.; IV MD, MSc, PhD. Adjunct Professor, Department of Orthopedics and Traumatology. Discipline of Hand and Upper Limb Surgery, Universidade Federal de São Paulo (UNIFESP), São Paulo (SP), Brazil.; V MD, MSc, PhD. Adjunct Professor, Department of Orthopedics and Traumatology, Discipline of Hand and Upper Limb Surgery, Escola Paulista de Medicina (EPM), Universidade Federal de São Paulo (UNIFESP), São Paulo (SP), Brazil.

**Keywords:** Diagnosis, Carpal tunnel syndrome, Randomized clinical trials [publication type], Systematic Review [publication type], Diagnostic, Outcome, Carpal tunnel, Surgery

## Abstract

**BACKGROUND:**

The diagnostic criteria for carpal tunnel syndrome (CTS) lack uniformity. Moreover, because CTS is a syndrome, there is no consensus as to which signs, symptoms, clinical and complementary tests are more reproducible and accurate for use in clinical research. This heterogeneity is reflected in clinical practice. Thus, establishing effective and comparable care protocols is difficult.

**OBJECTIVE:**

To identify the diagnostic criteria and outcome measures used in randomized clinical trials (RCTs) on CTS.

**DESING AND SETTING:**

Systematic review of randomized clinical trials carried out at the Federal University of São Paulo, São Paulo, Brazil.

**METHODS:**

We searched the Cochrane Library, PubMed, and Embase databases for RCTs with surgical intervention for CTS published between 2006 and 2019. Two investigators independently extracted relevant data on diagnosis and outcomes used in these studies.

**RESULTS:**

We identified 582 studies and 35 were systematically reviewed. The symptoms, paresthesia in the median nerve territory, nocturnal paresthesia, and special tests were the most widely used clinical diagnostic criteria. The most frequently assessed outcomes were symptoms of paresthesia in the median nerve territory and nocturnal paresthesia.

**CONCLUSION:**

The diagnostic criteria and outcome measures used in RCTs about CTS are heterogeneous, rendering comparison of studies difficult. Most studies use unstructured clinical criteria associated with ENMG for diagnosis. The Boston Questionnaire is the most frequently used main instrument to measure outcomes.

**REGISTRATION:**

PROSPERO (CRD42020150965- https://www.crd.york.ac.uk/prospero/display_record.php?RecordID=150965).

## INTRODUCTION

Carpal tunnel syndrome (CTS) is the most prevalent peripheral neuropathy in the world.^1,2^ Although some patients are treated conservatively, most require surgical treatment, which generates spending more than US$ 2 billion/year.^2^

The socioeconomic impact of the disease drove numerous randomized clinical trials (RCTs) to determine the best treatment for CTS. To identify effective interventions, accurate and relevant outcomes for the patient are needed.^3–6^ There is extensive literature about objective outcomes (e.g., loss of strength) and variables derived from nerve conduction studies. However, how these outcomes translate into tangible benefits to the patients remains unclear.^3–6^

In CTS, the lack of uniform criteria poses a challenge in diagnosis. Thus, Graham proposed well-defined criteria, based on a robust methodology.^7^ Moreover, because CTS is a syndrome, experts do not agree on which signs, symptoms, clinical and complementary tests are more reproducible and accurate in clinical research.^8^

This heterogeneity is reflected in clinical practice and has led to difficulty in establishing effective and comparable care protocols.^9,10^ Thus, studies must use precise diagnostic methods and clarify the main post-surgical outcomes to be evaluated in patients with CTS.

Systematic reviews promote synthesis, provide a comprehensive view, and recommend the best available evidence on a topic. Diagnostic and rational management criteria are of interest.^8,10^ Importantly, evaluation outcomes should reflect the impact of treatments on body structure and function, including activity limitations and participation restrictions, through a broad evaluation model.^11–13^ The Classification of Functioning and Disability and Health (ICF), approved in 2001 by the World Health Organization, proposed a comprehensive assessment from both individual and social perspectives.^14,15^ The model aimed to recognize the abnormalities in the body structure, identify the consequences on function, and describe the repercussions and adaptations to such changes in the individual's social dynamics.^14^ A previous systematic review on the subject was published in 2006.^8^ Given the importance of the topic, we sought to give an update.

## OBJECTIVE

The objective of this systematic review is to compare the diagnostic criteria and outcome measures based on ICF used in CTS over the past 15 years.

## METHODS

This systematic review was approved by the Research Ethics Committee (no. 2248181019) and developed in accordance with the Preferred Reporting Items for Systematic Reviews and Meta-Analyses (PRISMA) statement. The protocol was published a priori in the PROSPERO database (CRD42020150965 - https://www.crd.york.ac.uk/prospero/display_record.php?RecordID=150965).

### Search strategy

We conducted a search of works published in English, from 2006 to 2019, at the Cochrane Library, Medline (via PubMed), and Embase (via Ovid). The search was performed independently by RLS and AFZ. We use the terms: carpal tunnel syndrome and randomized controlled trial along with the Boolean term AND for free search of Cochrane Library and Embase. For Medline, we searched the MeSH term carpal tunnel syndrome and randomized controlled trial; we then used the PubMed Search Builder tool to combine terms.

### Criteria for selection of studies and procedures

After the initial screening based on the title and abstract, the full-text articles were independently reviewed by RLS and AFZ. These were included if they met the eligibility criteria enumerated below. Disagreements were resolved by a third author, VYM.

The inclusion criteria were: 1. Type of study: randomized clinical trials with follow-up longer than three months; 2. Patients: adults (>18 years) with initial diagnosis of CTS.

### Exclusion criteria

1. Studies not published in English.
2. Studies that did not involve at least one surgical intervention

### Data extraction

We extracted the following data: 1. Study design (country and year of publication); 2. Experimental and control interventions; 3. Sample size; 4. Follow-up time; 5. Blinding; 6. Diagnostic criteria; 7. Pre- and post-operative outcome measures.

### Methodological quality assessment

We use the Cochrane Collaboration Risk-of-Bias tool,^51^ which evaluates: 1. random sequence generation (selection bias); 2. Allocation Concealment (selection bias); 3. Blinding of participants and staff (performance bias); 4. Blinding of assessments and outcome (detection bias); 5. Incomplete outcomes (friction bias); 6. Selective outcome reporting (reporting bias) and 7. Other sources of bias (other biases).

### Assessment of outcomes based on the International Classification of Functioning, Disability and Health (ICF)

This classification facilitates understanding of health determinants and health-related effects through a standardized and comprehensive terminology.^15^ Correlating the pathophysiology of CTS with its clinical manifestations (i.e., signs and symptoms) assists in identifying specific structures and functions of the body altered by the disease (first domain of ICF). Additionally, patients may also have disabilities or limitations in performing activities of daily life (second domain of ICF), which impact situations of social life and satisfaction (third domain of ICF).^8,14^

### Data analysis

The data collected were presented in tables. Each study was labelled according to its author. The data was managed in Excel 2020 (Microsoft Corporation, Redmond, Washington, United States).

## RESULTS

**Figure 1:**
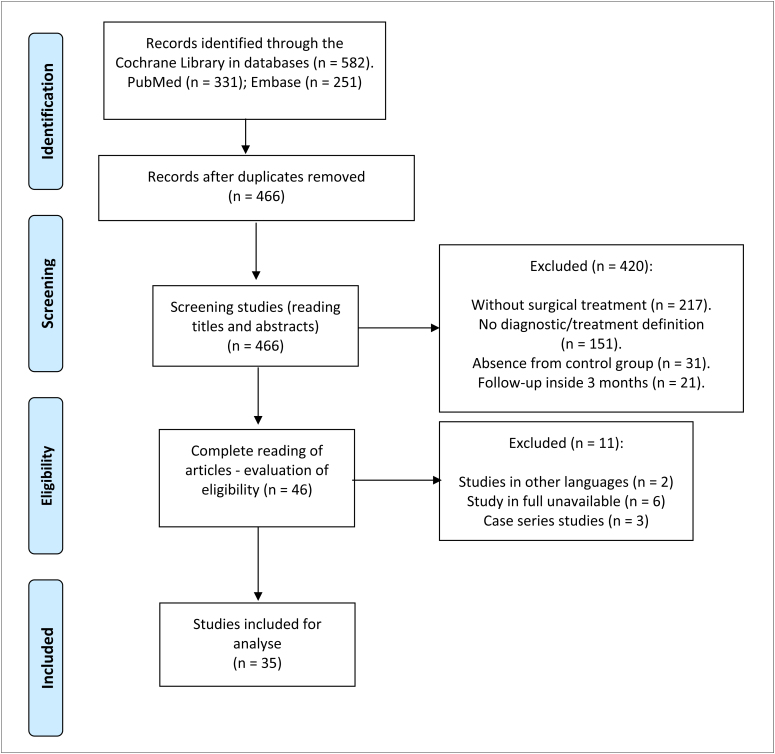
Flow diagram of eligible studies.^16–50^

From the 582 studies screened, 35 were included in the systematic review (Figure 1).^16–50^ Table 1 provides a meta-summary of the characteristics of the studies included.^16–50^

**Table 1:** Summary of the included randomized controlled trials

First Author	Country	Diagnostic Criteria		Experimental studies	Intervention Control	Sample Size		Follow-up (months)	Blinding
		Clinics	Complementary examinations			Patients	Hands		
Rab^16^	Austria	Paresthesia + special tests	ENMG (Non-Padua criteria)	Endoscopic	Classic open CTD release	10	20	12	
Siegmeth^17^	United Kingdom	Paresthesia + special tests	–	CTD open modified release	Classic open CTD release	42	84	6	x
Zyluk^18^	Poland	Unspecified	ENMG (Non-Padua criteria)	CTD open modified release	CTD open modified release	65	73	12	x
Forward^19^	United Kingdom	Graham Criteria (CTS-6)	ENMG (Non-Padua criteria)	Classic open CTD release	CTD open modified release	112	112	3	x
Atroshi^20^	Sweden	Diagram Katz	ENMG (Non-Padua criteria)	Endoscopic	Classic open CTD release	128	128	12	x
Huemer^21^	Austria	Unspecified	ENMG (Non-Padua criteria)	Classic open CTD + release Small bandage	Classic open CTD release + Bulky bandage	50	50	3	
Pomerance^22^	United States	Paresthesia + special tests	ENMG (Non-Padua criteria)	Classic open CTD release + Physiotherapy	Classic open CTD release	150	150	6	x
Atroshi^23^	Sweden	Katz Diagram	ENMG (Non-Padua criteria)	Endoscopic	Classic open CTD release	126	126	60	
Gordon^24^	Canada	Unspecified	ENMG (Non-Padua criteria)	Physiotherapy	Classic open CTD release	21	21	12	
Faraj^25^	Iraq	Paresthesia + special tests	ENMG (Non-Padua criteria)	CTD open modified release	Classic open CTD release	40	40	3	
Nabhan^26^	Germany	Unspecified	ENMG (Non-Padua criteria)	Classic open CTD release + local anaesthesia	Classic open CTD release + regional anesthesia	43	43	6	x
Eriji^27^	Japan	Paresthesia + special tests	ENMG (Non-Padua criteria)	Endoscopic	Open Surgery	79	101	3	x
Uçar^28^	Turkey	Unspecified	ENMG (Non-Padua criteria)	CTD open modified release	CTD open modified release	90	90	30	
Larsen^29^	Denmark	Unspecified	ENMG (Non-Padua criteria)	CTD open modified release	Endoscopic	90	90	6	x
Tarallo^30^	Italy	Paresthesia + special tests	ENMG (Non-Padua criteria)	CTD open modified release	Classic open CTD release	120	120	12	
Ullah^31^	Pakistan	Paresthesia + special tests	ENMG (Non-Padua criteria)	Classic open CTD release	Pharmacological	40	40	13	x
Andreu^32^	Spain	Katz Diagram	ENMG (Non-Padua criteria)	Classic open CTD release	Pharmacological	95	95	12	
Vanni^33^	Italy	Paresthesia + special tests	ENMG (Non-Padua criteria)	CTD open modified release	Classic open CTD release	220	220	12	x
Peñas^34^	Spain	Paresthesia + special tests	ENMG (Non-Padua criteria)	Physiotherapy	Classic open CTD release	111	111	12	x
SadatsuneI^35^	Brazil	Paresthesia + special tests	ENMG (Non-Padua criteria)	Classic open CTD release + Pharmacological	Classic open CTD release + Pharmacological	37	37	6	x
Rojo-Manaute^36^	Arab Emirates	Unspecified	ENMG (Non-Padua criteria)	CTD open modified release	CTD open modified release	82	82	12	x
Acar^37^	Turkey	Paresthesia + special tests	ENMG (Non-Padua criteria)	Classic open CTD release	CTD open modified release	113	159	24	x
Gumustas^38^	France	Unspecified	ENMG (Non-Padua criteria)	Endoscopic	Classic open CTD release	50	50	–	x
Cho^39^	South Korea	Paresthesia + special tests	ENMG (Non-Padua criteria)	CTD open modified release	CTD open modified release	79	79	24	x
Herold^40^	United Kingdom	Paresthesia + special tests	–	Physiotherapy	Classic open CTD release	93	93	3	x
Peñas^41^	Spain	Paresthesia + special tests	ENMG (Non-Padua criteria)	Classic open CTD release	Physiotherapy	120	120	12	x
Logli^42^	United States	Unspecified	ENMG (Non-Padua criteria)	Physiotherapy	Classic open CTD release	249	249	12	x
Peñas^43^	Spain	Paresthesia + special tests	ENMG (Non-Padua criteria)	Classic open CTD release	Physiotherapy	100	100	12	x
Peñas^44^	Spain	Paresthesia + special tests	ENMG (Non-Padua criteria)	Physiotherapy	Classic open CTD release	95	95	12	x
Boriani^45^	Italy	Paresthesia + special tests	ENMG (Non-Padua criteria)	Classic open CTD release + Pharmacological	Classic open CTD release + Pharmacological	64	64	3	x
Kleermaeker^46^	Germany	Paresthesia + special tests	ENMG (Non-Padua criteria)	Classic open CTD release	Physiotherapy and Pharmacology	43	43	6	x
Kanchanathepsak^47^	Thailand	Unspecified	–	CTD open modified release	Classic open CTD release	33	36	3	x
Oh^48^	South Korea	Unspecified	Ultrasonography	Endoscopic	CTD open modified release	67	67	6	
Rimdeika^49^	Germany	Unspecified	ENMG (Non-Padua criteria)	CTD open modified release	Classic open CTD release	104	104	4	
Zhang^50^	China	Paresthesia + special tests	ENMG (Non-Padua criteria)	CTD open modified release	Pharmacological	46	46	3	x
TOTAL n = 35						**3,007**	**3,138**		
Average ± SD						**82 ± 51**	**90 ± 51**	**12 ± 11**	

CDT = carpal transverse ligament; ENMG = electroneuromyography; SD = standard deviation.

The outcome measures reported in the RCTs were classified according to the domains of the ICF: A) Body functions and structures (Table 2);^16–50^ B) Activity limitations (Table 3)^16–50^ and C) Social life/Satisfaction (Table 3).^16–50^

**Table 2:** Outcomes in randomized clinical trials - body functions and structures

First Author	Symptoms	Motor Functions			Sensitive Functions			Body Structures			
	Paresthesia in the territory of the median and nocturnal nerve	Manual Grasping Force	Tweezers	Pick-up Test	2 Point Discrimination	Monofilament	Vibration	Nerve Conduction (sensitive and motor)	Wound Complications	Local Sensitivity Disorders	Causalgia
	Standard questionnaire used										
Rab^16^	Boston Questionnaire	x	x		x	x		x			
Siegmeth^17^	PEM score	x								x	
Zyluk^18^	Boston Questionnaire	x	x		x	x					
Forward^19^	PEM score	x	x								
Atroshi^20^	Boston Questionnaire	x	x		x	x				x	
Huemer^21^	–	x		x	x			x		x	
Pomerance^22^	Dash score	x	x								
Atroshi^23^	–										
Gordon^24^	Boston Questionnaire						x	x			
Faraj^25^	–								x		
Nabhan^26^	MHQ										
Eriji^27^	–	x	x		x	x		x		x	
Uçar^28^	Boston Questionnaire										
Larsen^29^	–	x									
Tarallo^30^	Boston Questionnaire	x	x		x				x	x	
Ullah^31^	–										
Andreu^32^	–								x		
Vanni^33^	Boston Questionnaire										
Peñas^34^	–							x			
SadatsuneI^35^	–										x
Rojo-Manaute^36^	Quickdash score	x								x	
Acar^37^	–							x		x	
Gumustas^38^	Boston Questionnaire							x			
Cho^39^	Boston Questionnaire									x	
Herold^40^	MHQ			x	x	x	x			x	
Peñas^41^	Boston Questionnaire										
Logli^42^	Dash score	x							x		
Peñas^43^	Boston Questionnaire										
Peñas^44^	Boston Questionnaire		x								
Boriani^45^	Boston Questionnaire				x			x			
Kleermaeker^46^	Boston Questionnaire										
Kanchanathepsak^47^	Boston Questionnaire	x	x		x	x		x	x		
Oh^48^	Boston Questionnaire + Dash score										
Rimdeika^49^	Dash score	x	x		x	x					
Zhang^50^	Boston Questionnaire							x			

PEM score = Patient Evaluation Measure; Dash = Disabilities of the Arm, Shoulder and Hand; MHQ = Michigan Hand Outcomes Questionnaire.

**Table 3:** Outcomes in randomized clinical trials - activity and social life limitations/satisfaction

First author	Activities (limitations)			Social life/Satisfaction		
	Dexterity	Use of hands in AVD's	Functional Status Scale - Boston Questionnaire	Time away from work	Satisfaction	Aesthetics
Rab^16^			Applied			
Siegmeth^17^		x	Not applied			x
Zyluk^18^			Applied			
Forward^19^			Not applied			
Atroshi^20^		x	Applied	x		
Huemer^21^			Not applied			
Pomerance^22^			Not applied	x		
Atroshi^23^			Not applied		x	
Gordon^24^	x		Applied			
Faraj^25^			Not applied		x	
Nabhan^26^		x	Not applied	x	x	x
Eriji^27^		x	Not applied			
Uçar^28^			Applied			
Larsen^29^			Not applied			
Tarallo^30^			Applied		x	x
Ullah^31^			Not applied			
Andreu^32^		x	Not applied			
Vanni^33^			Applied			x
Peñas^34^			Not applied			
SadatsuneI^35^			Not applied			
Rojo-Manaute^36^			Not applied	x		
Acar^37^			Not applied			
Gumustas^38^		x	Applied			
Cho^39^			Applied		x	
Herold^40^	x	x	Not applied			
Peñas^41^		x	Applied		x	
Logli^42^			Not applied			
Peñas^43^			Applied			
Peñas^44^			Applied			
Boriani^45^			Applied			
Kleermaeker^46^			Applied			
Kanchanathepsak^47^			Applied			
Oh^48^			Applied			
Rimdeika^49^		x	Not applied			
Zhang^50^			Applied			

### Characteristics of studies and evaluated outcomes

We analyzed studies that evaluated the effectiveness of different surgical and conservative techniques; some studies used more than one intervention. In the experimental group, classical open carpal ligament (CLL) release (12; 34%), modified open CLL release (12; 34%) and endoscopic CT release (6; 17%) were used. In addition, conservative interventions such as physiotherapy (5; 14%) and the use of drugs (3; 8%) were also tested. As control, classical open CT release (20; 57%), modified open CT release (7; 20%), endoscopic CT release (1; 3%), open surgery (1; 3%), physiotherapy (3; 8%) and drugs (6; 17%) were used. A total of 3,007 patients and 3138 hands were studied (some patients received treatments for both hands). The follow-up time ranged from 3 to 60 months. The average follow-up was 12 months; five reported follow-up longer than 13 months. From the total, 25 studies (71%) showed adequate blinding.

The studies analyzed the following clinical diagnostic criteria for CTS: paresthesia in the territory of the median nerve, night paresthesia, and Phalen's and Tinel's tests (part of the six criteria described by Graham) (18 studies; 51%), the Katz diagram (3; 9%) and all the Graham criteria - CTS-6 (2; 6%). Other studies (12; 34%) did not specify the diagnostic method used (Table 1). Studies that used only part of the six criteria described by Graham^7^ were classified as paresthesia and special tests.

Electroneuromyography (ENMG) was a complementary examination in 31 studies (89%) and ultrasonography in only one (3%). The studies that used ENMG were then classified based on the use of the Padua criteria,^52^ used by 22 (71%). Three other studies (9%) did not use any type of complementary examination (Table 1).^16–50^

### Diagnostic criteria

#### Risk of bias - Cochrane Collaboration

**Figure 2:**
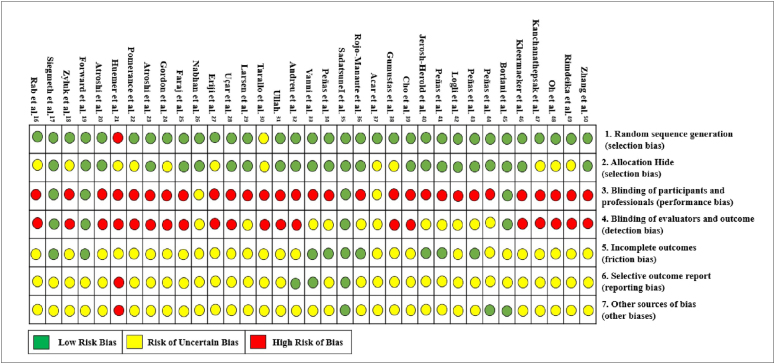
Risk of bias of randomized clinical trials included in the study - Cochrane Collaboration Tool.

Figure 2 presents the risk of study bias.^16–50^ Because surgical intervention was involved, blinding was difficult; most were classified as having uncertain or high risk of bias.

#### Categorization of the outcomes analyzed based on the International Classification of Functionality, Disability and Health (ICF)

The outcomes reported in the ECR were categorized according to the three domains of the ICF.

**A) Body functions and structures** (Table 2): Among the outcomes analyzed, symptoms (paresthesia in the territory of the median and nocturnal nerve) were the most frequently employed (26 studies; 74%).^16–50^

Standardized questionnaires were used in 25 studies (71%): the Boston Questionnaire (BQ) (17; 65%), Disabilities of the Arm, Shoulder and Hand (Dash score) (4; 15%), Patient Evaluation Measure (PEM score) (2; 8%), Michigan Hand Outcomes Questionnaire (MHQ) (2; 8%), and QuickDash score (1; 4%). Only one study used more than one questionnaire.

Motor functions, included in 16 studies (46%), were operationally defined as manual grasping force (14; 88%), tweezers (10; 62%) and pick-up test (2; 12%).

Sensory function was evaluated in 11 studies (31%). The most studied variable was two-point discrimination (10; 91%), followed by the monofilament test (8; 73%) and vibration (1; 10%).

Finally, the body structures were analyzed in 18 studies (51%), through sensory and motor nerve conduction (12; 67%), local sensitivity disorders (9; 50%), wound complications (3; 17%) and causalgia (1; 6%).

**B) Limitations of activity** (Table 3): Twenty-four (69%) studies evaluated activity limitations. The functional status scale of the BQ was the most frequently used outcome (17; 71%). The use of hands in daily life activities was analyzed in nine (38%) and dexterity in only two studies (8%).^16–50^

**C) Restrictions of activities of social life/satisfaction** (Table 3): Participation restrictions were analyzed in 12 studies (34%). Satisfaction was the most frequent outcome (6; 50%), followed by time off work (4; 33%) and aesthetic (4; 33%).^16–50^

## DISCUSSION

This systematic review mapped the diagnostic criteria and outcome measures used in CTS ECRs. Paresthesia, in conjunction with special tests (part of Graham's criteria)^7^, was the most widely used diagnostic clinical criterion, together with the complementary ENMG examination (mostly without the use of structured classification, such as that of Padua).^52^ Various outcome measures were found; these categorized according to the domains of the ICF. For body functions and structures, symptoms (paresthesia in the territory of the median and nocturnal nerve) were the most evaluated outcomes, measured predominantly by means of BQ. The functional status scale of the BQ was the outcome of the highest evaluation in assessing activity limitations. Finally, participation/satisfaction restrictions were mainly evaluated through patient satisfaction.

Research for diagnostic methods (clinical and complementary) of CTS is important because of the high prevalence and potential disability resulting from the disease.^1,2^ The presence of classical signs and symptoms (numbness and tingling in the distribution of the median nerve with nocturnal worsening) is appropriate for the diagnosis in most patients,.^53^ However, clinical and complementary tests are important in most cases to determine the suitability of surgical or conservative management.^53^ Graham's criteria are widely recommended.^7^ However, systematic reviews challenge the use of two-point discrimination (one of Graham's criteria), due to its low diagnostic sensitivity for CTS.^54^ Our results suggest the same, because most of the studies do not use two-point discrimination.

ENMG is widely used as a complementary quantitative method and is considered an important tool for analyzing and monitoring CTS intensity.^52,54,55^ Few studies utilized ENMG to predict outcomes for CTS.^52,55,56^ The ENMG Padua criteria (Electroneuromyography classification for stratification of median nerve involvement in CTS), is one of the most widely used tools.^52^ However, although ENMG was a predominant complementary examination in the included studies, most did not use the quantitative criteria of Padua fully.

In addition to effective diagnostic methods, the correct definition of primary and secondary outcomes in RCTs allows the generation of responses to the hypotheses previously defined in these studies.^8,10^ The focus of the included studies was the outcomes of body function and structure, with less attention to activity limitation and participation restriction. BQ was the most widely used, being an important outcome measure for assessing symptoms (body function and structure) and functional capacity (activity limitations). BQ has good psychometric properties in patients with CTS.^57–59^ Thus, its use should replace other non-standardized methods.^59^

Similarly, previous systematic reviews were less focused on outcomes related to activity limitations and participation restriction.^8,60^ Gummesson et al. reviewed 92 studies of upper limb dysfunction. The authors demonstrated that the outcomes of body function and structure were used in all studies, while only 41% of these also used measures of activity and participation.^60^

Jerosch-Herold et al.,^8^ investigated the most valid and accurate tools to assess the clinical outcomes of the CTS. The authors also reported that most of the variables evaluated (sensory functions, pain sensations, motor functions and sleep functions), were concentrated in body functions and structures. Outcomes related to activity limitations and participation restrictions were evaluated less frequently and included the functional status scale of the BQ, timed manual dexterity test, and reported time to resume activities of daily living. The only participation restriction measures were the number of days to return to work and patient satisfaction.

Considering these findings, our review informs the selection of precise outcomes for future CTS ECRs and highlights the most utilized clinical and complementary diagnostic instruments. Future RCTs should use paresthesia in the median nerve territory, nocturnal paresthesia, and special tests (i.e., the Phalen's and Tinel's tests), and ENMG with quantitative Padua criteria as diagnostic criteria for CTS. To reflect the impact of treatment on the three domains of analysis of ICF (body functions and structures, activity limitations and participation restrictions), BQ and participation restriction measures (e.g., number of days to return to work and patient satisfaction) should be standardized as main outcomes of analysis.

This is the first systematic review aimed at identifying the diagnostic criteria and the outcome measures used in ECR on CTS. The protocol was previously published in the PROSPERO database, restricting biases in methodology and enhancing credibility.^6^ In addition, in order to improve the quality of the report of this systematic review, the PRISMA statement was used.

Our review has several limitations. We only looked for studies written in English. Because we eliminated studies with less than three months follow-up to eliminate anesthesiology papers, studies of surgical interest may have been lost. We considered only RCTs, due to the greater ability to identify of the outcome. However, longitudinal studies also report results of surgical processes, and their non-inclusion may have generated the loss of important outcome and diagnostic measures.

## CONCLUSION

Almost half of the high-level methodological studies do not support diagnosis based on structured clinical criteria, such as Graham's. Most use ENMG as a complementary examination. Contrary to the literature, most studies do not prioritize patient-reported outcomes as relevant or primary outcomes. A task force is needed to standardize CTS research.

## References

[1] Dale AM, Harris-Adamson C, Rempel D (2013). Prevalence and incidence of carpal tunnel syndrome in US working
populations: pooled analysis of six prospective studies. Scand J Work Environ Health.

[2] Foley M, Silverstein B, Polissar N (2007). The economic burden of carpal tunnel syndrome: long-term earnings
of CTS claimants in Washington State. Am J Ind Med.

[3] Eriksen MB, Frandsen TF (2018). The impact of patient, intervention, comparison, outcome (PICO)
as a search strategy tool on literature search quality: a systematic
review. J Med Libr Assoc.

[4] Yordanov Y, Dechartres A, Ravaud P (2018). Patient-important outcomes in systematic reviews: Poor quality of
evidence. PLoS One.

[5] de Moraes VY, Ferrari PM, Gracitelli GC, Faloppa F, Belloti JC (2014). Outcomes in orthopedics and traumatology: translating research
into practice. Acta Ortop Bras.

[6] Belloti JC, Okamura A, Scheeren J, Faloppa F, Ynoe de Moares V (2019). A systematic review of the quality of distal radius systematic
reviews: Methodology and reporting assessment. PLoS One.

[7] Graham B, Regehr G, Naglie G, Wright JG (2006). Development and validation of diagnostic criteria for carpal
tunnel syndrome. J Hand Surg Am.

[8] Jerosch-Herold C, Leite JC, Song F (2006). A systematic review of outcomes assessed in randomized controlled
trials of surgical interventions for carpal tunnel syndrome using the
International Classification of Functioning, Disability and Health (ICF) as
a reference tool. BMC Musculoskelet Disord.

[9] Pantaleon L (2019). Why measuring outcomes is important in health
care. J Vet Intern Med.

[10] Clarke M, Williamson PR (2016). Core outcome sets and systematic reviews. Syst Rev.

[11] Castaneda L, Bergmann A, Bahia L (2014). The International Classification of Functioning, Disability and
Health: a systematic review of observational studies. Rev Bras Epidemiol.

[12] Stucki G, Bickenbach J (2017). Functioning: the third health indicator in the health system and
the key indicator for rehabilitation. Eur J Phys Rehabil Med.

[13] Castaneda L (2018). International Classification of Functioning, Disability and
Health (ICF) – way to Health Promotion. Rev Bras Cineantropom Desempenho Hum.

[14] Jiménez Buñuales MT, González Diego P, Martín Moreno JM (2002). La clasificación internacional del funcionamiento de la
discapacidad y de la salud (CIF) 2001 [International classification of
functioning, disability and health (ICF) 2001]. Rev Esp Salud Publica.

[15] Farias N, Buchalla CM (2005). A classificação internacional de funcionalidade, incapacidade e
saúde da organização mundial da saúde: conceitos, usos e
perspectivas. Rev Bras Epidemiol.

[16] Rab M, Grünbeck M, Beck H (2006). Intra-individual comparison between open and 2-portal endoscopic
release in clinically matched bilateral carpal syndrome. J Plast Reconstr Aesthet Surg.

[17] Siegmeth AW, Hopkinson-Woolley JA (2006). Standard open decompression in carpal tunnel syndrome compared
with a modified open technique preserving the superficial skin nerves: a
prospective randomized study. J Hand Surg Am.

[18]  Zyluk A, Strychar J (2006). A comparison of two limited open techniques for carpal tunnel
release. J Hand Surg Br.

[19] Forward DP, Singh AK, Lawrence TM (2006). Preservation of the ulnar bursa within the carpal tunnel: does it
improve the outcome of carpal tunnel surgery? A randomized, controlled
trial. J Bone Joint Surg Am.

[20] Atroshi I, Larsson GU, Ornstein E (2006). Outcomes of endoscopic surgery compared with open surgery for
carpal tunnel syndrome among employed patients: randomised controlled
trial. BMJ.

[21] Huemer GM, Koller M, Pachinger T (2007). Postoperative splinting after open carpal tunnel release does not
improve functional and neurological outcome. Muscle Nerve.

[22] Pomerance J, Fine I (2007). Outcomes of carpal tunnel surgery with and without supervised
postoperative therapy. J Hand Surg Am.

[23] Atroshi I, Hofer M, Larsson GU (2009). Open compared with 2-portal endoscopic carpal tunnel release: a
5-year follow-up of a randomized controlled trial. J Hand Surg Am.

[24] Gordon T, Amirjani N, Edwards DC, Chan KM (2010). Brief post-surgical electrical stimulation accelerates axon
regeneration and muscle reinnervation without affecting the functional
measures in carpal tunnel syndrome patients. Exp Neurol.

[25] Faraj AA, Ahmed MH, Saeed OA (2012). A comparative study of the surgical management of carpal tunnel
syndrome by mini-transverse wrist incisions versus traditional longitudinal
technique. Eur J Orthop Surg Traumatol.

[26] Nabhan A, Steudel WI, Dedeman L, Al-Khayat J, Ishak B (2011). Subcutaneous local anesthesia versus intravenous regional
anesthesia for endoscopic carpal tunnel release: a randomized controlled
trial. J Neurosurg.

[27] Ejiri S, Kikuchi S, Maruya M (2012). Short-term results of endoscopic (Okutsu method) versus palmar
incision open carpal tunnel release: a prospective randomized controlled
trial. Fukushima J Med Sci.

[28] Uçar BY, Demirtaş A, Bulut M, Azboy I, Uçar D (2012). Carpal tunnel decompression: two different mini-incision
techniques. Eur Rev Med Pharmacol Sci.

[29] Larsen MB, Sorensen AI, Crone KL, Weis T, Boeckstyns ME (2013). Carpal tunnel release: a randomized comparison of three surgical
methods. J Hand Surg Eur Vol.

[30] Tarallo M, Fino P, Sorvillo V, Parisi P, Scuderi N (2014). Comparative analysis between minimal access versus traditional
accesses in carpal tunnel syndrome: a perspective randomised
study. J Plast Reconstr Aesthet Surg.

[31] Ullah I (2013). Local steroid injection or carpal tunnel release for carpal
tunnel syndrome - which is more effective?. J Postgrad Med Inst.

[32] Andreu JL, Ly-Pen D, Millán I, de Blas G, Sánchez-Olaso A (2014). Local injection versus surgery in carpal tunnel syndrome:
neurophysiologic outcomes of a randomized clinical trial. Clin Neurophysiol.

[33]  Vanni D, Sirabella FS, Galzio R, Salini V, Magliani V (2015). The double tunnels technique: an alternative minimally invasive
approach for carpal tunnel syndrome. J Neurosurg.

[34]  Fernández-de-Las Peñas C, Ortega-Santiago R, de la Llave-Rincón AI (2015). Manual Physical Therapy Versus Surgery for Carpal Tunnel
Syndrome: A Randomized Parallel-Group Trial. J Pain.

[35] Sadatsune EJ, Leal Pda C, Cossetti RJ, Sakata RK (2016). Effect of preoperative gabapentin on pain intensity and
development of chronic pain after carpal tunnel syndrome surgical treatment
in women: randomized, double-blind, placebo-controlled study. Sao Paulo Med J.

[36] Rojo-Manaute JM, Capa-Grasa A, Chana-Rodríguez F (2016). Ultra-Minimally Invasive Ultrasound-Guided Carpal Tunnel Release:
A Randomized Clinical Trial. J Ultrasound Med.

[37] Acar MA, Kütahya H, Güleç A (2015). Triggering of the Digits After Carpal Tunnel
Surgery. Ann Plast Surg.

[38] Gümüştaş SA, Ekmekçi B, Tosun HB, Orak MM, Bekler Hİ (2015). Similar effectiveness of the open versus endoscopic technique for
carpal tunnel syndrome: a prospective randomized trial. Eur J Orthop Surg Traumatol.

[39]  Cho YJ, Lee JH, Shin DJ, Park KH (2016). Comparison of short wrist transverse open and limited open
techniques for carpal tunnel release: a randomized controlled trial of two
incisions. J Hand Surg Eur Vol.

[40] Jerosch-Herold C, Houghton J, Miller L, Shepstone L (2016). Does sensory relearning improve tactile function after carpal
tunnel decompression? A pragmatic, assessor-blinded, randomized clinical
trial. J Hand Surg Eur Vol.

[41] Fernández-de-Las-Peñas C, Cleland JA, Salom-Moreno J (2016). Prediction of Outcome in Women With Carpal Tunnel Syndrome Who
Receive Manual Physical Therapy Interventions: A Validation
Study. J Orthop Sports Phys Ther.

[42] Logli AL, Bear BJ, Schwartz EG, Korcek KJ, Foster BJ (2018). A Prospective, Randomized Trial of Splinting After Minicarpal
Tunnel Release. J Hand Surg Am.

[43] Fernández-de-Las-Peñas C, Cleland J, Palacios-Ceña M (2017). Effectiveness of manual therapy versus surgery in pain processing
due to carpal tunnel syndrome: A randomized clinical trial. Eur J Pain.

[44] Fernández-de-Las-Peñas C, Cleland J, Palacios-Ceña M (2017). The Effectiveness of Manual Therapy Versus Surgery on
Self-reported Function, Cervical Range of Motion, and Pinch Grip Force in
Carpal Tunnel Syndrome: A Randomized Clinical Trial. J Orthop Sports Phys Ther.

[45] Boriani F, Granchi D, Roatti G (2017). Alpha-lipoic Acid After Median Nerve Decompression at the Carpal
Tunnel: A Randomized Controlled Trial. J Hand Surg Am.

[46] De Kleermaeker FGCM, Meulstee J, Claes F, Kasius KM, Verhagen WIM (2017). Treatment outcome in patients with clinically defined carpal
tunnel syndrome but normal electrodiagnostic test results: a randomized
controlled trial. J Neurol.

[47] Kanchanathepsak T, Wairojanakul W, Phakdepiboon T (2017). Hypothenar fat pad flap vs conventional open release in primary
carpal tunnel syndrome: A randomized controlled trial. World J Orthop.

[48] Oh WT, Kang HJ, Koh IH, Jang JY, Choi YR (2017). Morphologic change of nerve and symptom relief are similar after
mini-incision and endoscopic carpal tunnel release: a randomized
trial. BMC Musculoskelet Disord.

[49]  Rimdeika R, Cepas A, Liubauskas R (2019). Proximal carpal crease incision for carpal tunnel release: a
pilot study. Eur J Plast Surg.

[50] Zhang S, Wang F, Ke S (2019). The Effectiveness of Ultrasound-Guided Steroid Injection Combined
with Miniscalpel-Needle Release in the Treatment of Carpal Tunnel Syndrome
vs. Steroid Injection Alone: A Randomized Controlled Study. Biomed Res Int.

[51] Higgins JP, Altman DG, Gøtzsche PC (2011). The Cochrane Collaboration's tool for assessing risk of bias in
randomised trials.

[52] Padua L, LoMonaco M, Gregori B (1997). Neurophysiological classification and sensitivity in 500 carpal
tunnel syndrome hands. Acta Neurol Scand.

[53] Keith MW, Masear V, Chung K (2009). Diagnosis of of carpal tunnel syndrome. J Am Acad Orthop Surg.

[54] Massy-Westropp N, Grimmer K, Bain G (2000). A systematic review of the clinical diagnostic tests for carpal
tunnel syndrome. J Hand Surg Am.

[55] Sonoo M, Menkes DL, Bland JD, Burke D (2018). Nerve conduction studies and EMG in carpal tunnel syndrome: Do
they add value?. Clin Neurophysiol Pract.

[56] Fowler JR (2017). Nerve Conduction Studies for Carpal Tunnel Syndrome: Gold
Standard or Unnecessary Evil?. Orthopedics.

[57] Levine DW, Simmons BP, Koris MJ (1993). A self-administered questionnaire for the assessment of severity
of symptoms and functional status in carpal tunnel syndrome. J Bone Joint Surg Am.

[58] Katz JN, Punnett L, Simmons BP (1996). Workers’ compensation recipients with carpal tunnel syndrome: the
validity of self-reported health measures. Am J Public Health.

[59] Leite JC, Jerosch-Herold C, Song F (2006). A systematic review of the psychometric properties of the Boston
Carpal Tunnel Questionnaire. BMC Musculoskelet Disord.

[60] Gummesson C, Atroshi I, Ekdahl C (2004). The Quality of reporting and outcome measures in randomized
clinical trials related to upper-extremity disorders. J Hand Surg Am.

